# Study on the changes and significance of CXCL10 level in serum of isolated polymyalgia rheumatica

**DOI:** 10.1007/s10067-024-07209-7

**Published:** 2024-10-30

**Authors:** X. U. Shuai, FENG Dandan, X. U. Liang

**Affiliations:** Department of Rheumatology and Immunology, The First Affiliated Hospital of Wannan Medical College, Wuhu, China

**Keywords:** CXCL10, Chemokines, Isolated polymyalgia rheumatica

## Abstract

**Objective:**

To investigate the significance of CXC chemokine ligand 10 (CXCL10) in the pathogenesis of isolated polymyalgia rheumatica (PMR).

**Methods:**

The serum of six PMR patients diagnosed and treated at the First Affiliated Hospital of Wannan Medical College from September 2019 to December 2020 before treatment and after remission was collected, and the serum of six active rheumatoid arthritis (RA) patients and six healthy medical checkups were also collected, and protein microarray technology was used to detect 24 cytokines, including IL-6, IL-4, CXCL10, CXCL8, and CXCL2. Subsequently, serum was collected from other 28 patients with active PMR, 26 patients with PMR in remission, 24 patients with active RA, and 24 healthy medical checkups who were diagnosed and treated at the First Affiliated Hospital of Wannan Medical College from January 2021 to July 2023, and the enzyme-linked immunosorbent assay (ELISA) was used to validate and compare the levels of CXCL10 in each group and analyze the correlation between the levels of serum CXCL10 and the parameters of the clinical activities of PMR.

**Results:**

Protein microarray screening revealed significant differences in CXCL10 before and after PMR treatment, and ELISA validation revealed that peripheral serum CXCL10 levels were significantly higher in the PMR-active group than in the remission group (*P* < 0.001), and also significantly higher than in the RA-active group (*P* = 0.003) and in the healthy control group (*P* < 0.001); correlation analysis showed a significant positive correlation between serum CXCL10 levels and serum ferritin in PMR patients (*r* = 0.450, *P* = 0.024). In the ROC curve for distinguishing PMR and RA, the area under the curve is 0.741, sensitivity = 0.643, and specificity = 0.792.

**Conclusion:**

CXCL10 may play a role in the pathogenesis of isolated PMR and its level might contribute to the differential diagnosis of PMR and RA.

**Table:** 

**Key Points** • *The concentration of CXCL10 was higher in peripheral blood of isolated PMR patients.* • *CXCL10 is a potential diagnostic biomarker for isolated PMR patients.* • *The level of CXCL10 might contribute to the differential diagnosis of PMR and RA.*

**Supplementary Information:**

The online version contains supplementary material available at 10.1007/s10067-024-07209-7.

## Introduction

Polymyalgia rheumatica (PMR) is a chronic inflammatory rheumatic disease characterized by pain and stiffness in the neck, shoulder, and pelvic girdle muscles in the elderly, which is complicated by giant cell arteritis in some patients, and by the presence of isolated rheumatic polymyalgia in others [1]. Studies have shown that the pro-inflammatory cytokine interleukin 6 (IL-6) plays an important role in the pathogenesis of isolated PMR [2]. However, the mechanisms that contribute to the persistence and progression of inflammation in PMR are unclear. Chemokines are small molecule proteins that have the role of inducing chemotaxis to a variety of cells. CXC Chemokine Ligand-10 (CXCL10) is a CXC family chemokine induced by interferon gamma (IFN-γ) and synthesized and secreted by a variety of immune cells (e.g., monocytes, macrophages, T cells, B cells, and NK cells) and non-immune cells (endothelial cells, epidermal cells, fibroblasts, etc.) [3]. CXCL10 regulates the immune response by binding to its receptor CXCR3 and activating and recruiting various immune cells such as T cells, eosinophils, monocytes, and NK cells [4, 5]. To investigate the role of chemokines in the pathogenesis of PMR, this study first screened for chemokines potentially associated with PMR pathogenesis by protein microarrays. In conjunction with previous studies in the literature which Van der Geest et al. found the significantly elevated serum CXCL10 levels in patients with active PMR [6]. CXCL10 was selected and an expanded sample size was used to validate altered serum CXCL10 levels in PMR versus control groups using enzyme immunoassay. The results of the study are reported below.

## Methods

### Patients

Six primary untreated PMR patients (PMR activity group) and six PMR patients in complete remission with treatment (PMR remission group) and six untreated RA patients (RA group) diagnosed at the Department of Rheumatology and Immunology, the First Affiliated Hospital of Wannan Medical College, Wannan Medical College, China, from September 2019 to December 2020 were selected, and six healthy medical checkups (healthy control group) were selected as the controls, and the serums of the groups were screened for differently expressed chemokines by using protein microarray technology. Then another 28 patients with active PMR and 26 patients with PMR in remission and 24 patients with active RA diagnosed and treated from January 2021 to July 2023 were collected; and 24 healthy medical check-ups as the normal control group, and the CXCL10 levels of each group were measured using ELISA. There was no significant difference in the age and sex ratios of the groups. All PMR patients’ data were obtained from our center’s registered research database (China Clinical Trial Registry registration number: ChiCTR1800019715). The diagnosis of PMR was in accordance with the 2012 European League Against Rheumatism (EULAR)/American College of Rheumatology (ACR) revised classification criteria [7] for PMR. And all PMR patients underwent arterial ultrasound examination to exclude subclinical GCA. The active PMR patients were untreated at the initial diagnosis or relapsed without treatment and the PMR-AS > 7 points. The PMR patients in remission were people who had no significant symptom and the level of ESR and CRP in normal and PMR-AS score less than 1.5. The diagnosis of RA was in accordance with the 1987 ACR and/or 2010 ACR/EULAR classification criteria [8] for RA. Exclusion criteria are as follows: (1) exclude patients with combined GCA, tumors, infections, severe liver and kidney dysfunction, etc. (2) Exclude patients with subclinical GCA or other connective tissue diseases during the follow-up process. The study was approved by the hospital ethics committee and all patients signed informed consent.

### Materials

Clinical data include age and gender. Laboratory examination including blood sedimentation rate (ESR), C-reactive protein (CRP), ferritin (sFe), and PMR activity score (PMR-AS) [9] was collected from patients with active PMR. PMR-AS = CRP (mg/dl) + VAS p (0–10) + VAS ph (0–10) + (MST (min) × 0.1) + EUL (3–0), (The visual analog scale for pain (VAS p), the visual analog scale for physician’s assessment (VAS ph), morning stiffness (MST), the ability to elevate the upper limbs (EUL)). EUL was assessed the way round (3 = none, 2 = below shoulder girdle, 1 = up to shoulder girdle, 0 = above shoulder girdle). A PMR-AS between 17 and 7 can be regarded as the range for medium disease activity, 17 for high, and 1.5–7 for low disease activity. A PMR-AS between 1.5 and 0 can be regarded as the remission [10]. Five milliliters of venous blood was collected aseptically from all subjects. After centrifugation, the supernatant was taken and stored in the refrigerator at − 80 ℃.

### Protein microarray screening for chemokines

Sera from six patients with PMR before and after treatment, six patients with primary rheumatoid arthritis, and six healthy medical examiners were screened using the chemokine Luminex liquid-phase suspension microarray method. Luminex liquid suspension chip detection was performed by Wayen Biotechnologies (Shanghai, China). The Bio-Plex Pro Human Chemokine Panel 40-plex kit was used in accordance with the manufacturer’s instructions.

### ELISA validation of the differently expressed chemokine CXCL10

The sera of 28 patients with active PMR, 26 patients with PMR in remission, 24 patients with active RA, and 24 healthy check-ups were screened for differently expressed CXCL10 by quantitative detection using ELISA, the Human CXCL10/IP-10 ELISA Kit (EK168-96) was purchased from Multi Sciences, and the operation was carried out in accordance with the instruction manual, and duplicate wells were set up for each sample.

### Statistical analysis

SPSS 25.0® statistical software was used for analysis. Measurements were first tested for normality, and those that met the normal distribution were described statistically using $\overline{X }$ ± *S*, and two independent samples *t*-test was used for comparison between groups; those that did not meet the normal distribution were described using the median (interquartile spacing) for statistical analysis, and Wilcoxon rank sum test was used for comparison between groups. Spearman correlation test and simple linear regression analysis were used to determine the correlation between the two variables, and the diagnostic and differential diagnostic efficacy is analyzed by receiver operating characteristic (ROC) curve. *P* < 0.05 was considered a statistical difference.

## Results

### Screening of cytokines in PMR serum by liquid-phase microarray technology

**Fig. 1:**
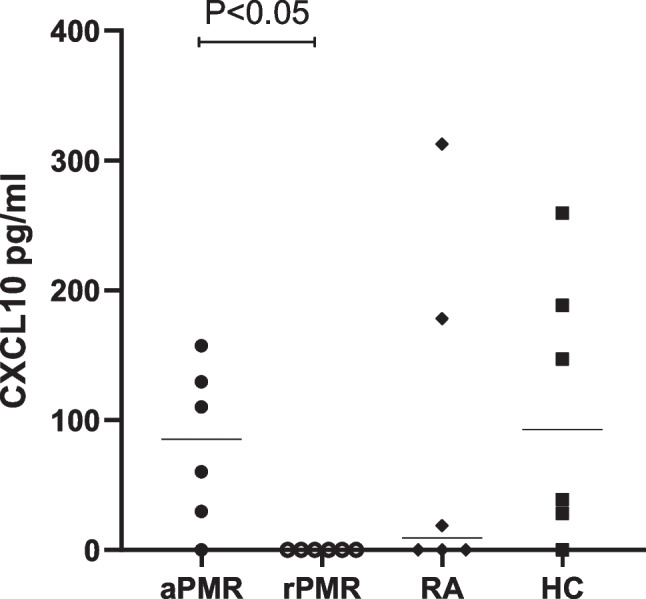
Protein microarray screening results for CXCL10. aPMR, active polymyalgia rheumatica; rPMR, remission polymyalgia rheumatica; RA, rheumatoid arthritis; HC, healthy controls

Sera from six active PMR patients and six PMR patients in remission after treatment, as well as six active RA patients and six normal controls, were tested by Luminex liquid-phase microarray technology. The results of Luminex liquid suspension chip are listed in the Sheet.1. The median serum level of CXCL10 was found to be 85.29 (IQR 37.35–124.66) pg/ml, in the active PMR group, which was significantly higher than that of 0.00 (IQR 0.00–0.00) pg/ml, in the remission PMR group, with a statistically significant difference (*Z* =  − 2.678, *P* = 0.007; Fig. 1). CXCL2 and CXCL8 in the serum of active PMR were also found higher than it in the remission PMR (*Z* =  − 2.082, *P* = 0.037; *Z* =  − 2.722, *P* = 0.006; Sheet.1). Serum CXCL10 levels in the active PMR group were not significantly different from those in the healthy control group at 92.92 (IQR 30.85–178.14) pg/ml, and in the RA group at 9.36 (IQR 0.00–138.38) pg/ml (*P* > 0.05).

### Validation of CXCL10 in PMR group by ELISA

**Fig. 2:**
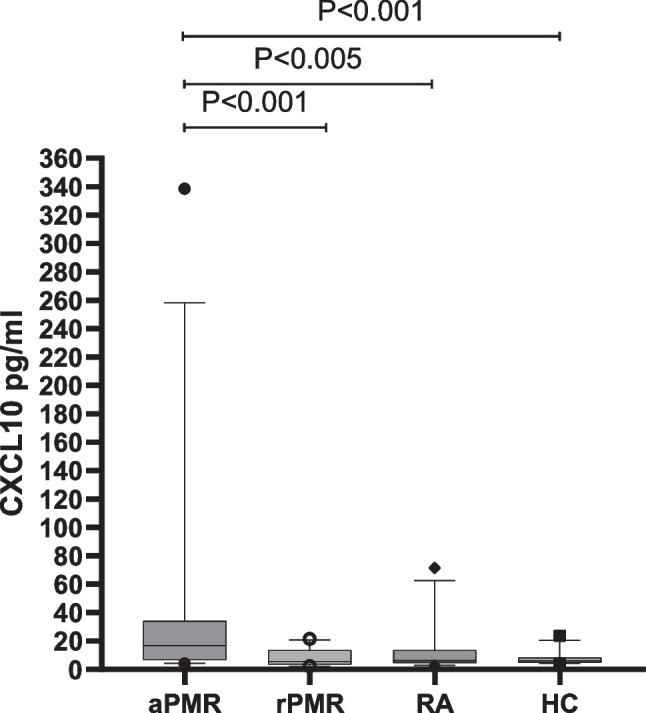
ELISA results for CXCL10. aPMR, active polymyalgia rheumatica; rPMR, remission polymyalgia rheumatica; RA, rheumatoid arthritis; HC, healthy controls

Serum was selected from an additional 28 active PMR patients, 26 remission PMR patients, 24 active RA patients, and 24 normal controls. And the serum levels of CXCL10 in each group were determined by ELISA. The median serum CXCL10 level was 16.92 (IQR 7.01–33.65) pg/ml, in the active PMR group, which was significantly higher than that of 6.26 (IQR 4.54–12.75) pg/ml, in the active RA group (*Z* =  − 2.974, *P* = 0.003), and also significantly higher than that of 5.56 (IQR 3.65–13.34) pg/ml, in the remission PMR group (*Z* =  − 3.670, *P* < 0.001) and 6.25 (IQR 4.84–8.12) pg/ml (*Z* =  − 3.662, *P* < 0.001) in the healthy control group, both of which were statistically significant differences. CXCL10 levels in the remission PMR group were not significantly different from those in the RA group (*Z* =  − 0.893, *P* = 0.372), nor from those in the healthy control group (*Z* =  − 0.660, *P* = 0.509), as shown in Fig. 2.

### Correlation analysis between clinical activity parameters and CXCL10 levels in patients with PMR

To find out whether the chemokine CXCL10 was correlated with the clinical activity of PMR, the correlation test was performed between CXCL10 levels and clinical activity indicators such as hs-CRP, ESR, and PMR-AS scores in 28 patients with active PMR. The results showed no significant correlation between CXCL10 levels and hs-CRP, ESR, and PMR-AS, as shown in Table 1. Three of the 28 PMR patients had missing ferritin data, and there was a significant positive correlation between serum CXCL10 level and ferritin in 25 active PMR patients (*r* = 0.450, *P* = 0.024), and 19 of the 25 active PMR patients had elevated ferritin levels outside of the normal range, while the remaining six were within the normal range, and there was still a statistically significant difference between the two ferritin levels (*Z* =  − 2.227, *P* = 0.026).

**Table 1:** Correlation of CXCL10 with clinical and laboratory data in the active PMR group. *ESR*, erythrocyte sedimentation rate; *CRP*, C reactive protein; *Fer*, ferritin; *WBC*, white blood cell; *RBC*, red blood cell; *Hb*, hemoglobin; *PLT*, blood platelet; *NEUT%*, the percentage of neutrophil; *LYM%*, the percentage of lymphocyte; *PMR-AS*, Polymyalgia Rheumatica Activity Scores

	ESR	CRP	Fer	WBC	RBC	Hb	PLT	NEUT%	LYM%	PMR-AS
*r*	− 0.144	0.242	0.450^*^	0.022	0.030	0.185	− 0.064	0.117	− 0.169	− 0.085
*P*	0.466	0.241	0.024	0.910	0.881	0.345	0.745	0.555	0.391	0.666

### The value of CXCL10 in the diagnosis and differential diagnosis of PMR

**Fig. 3:**
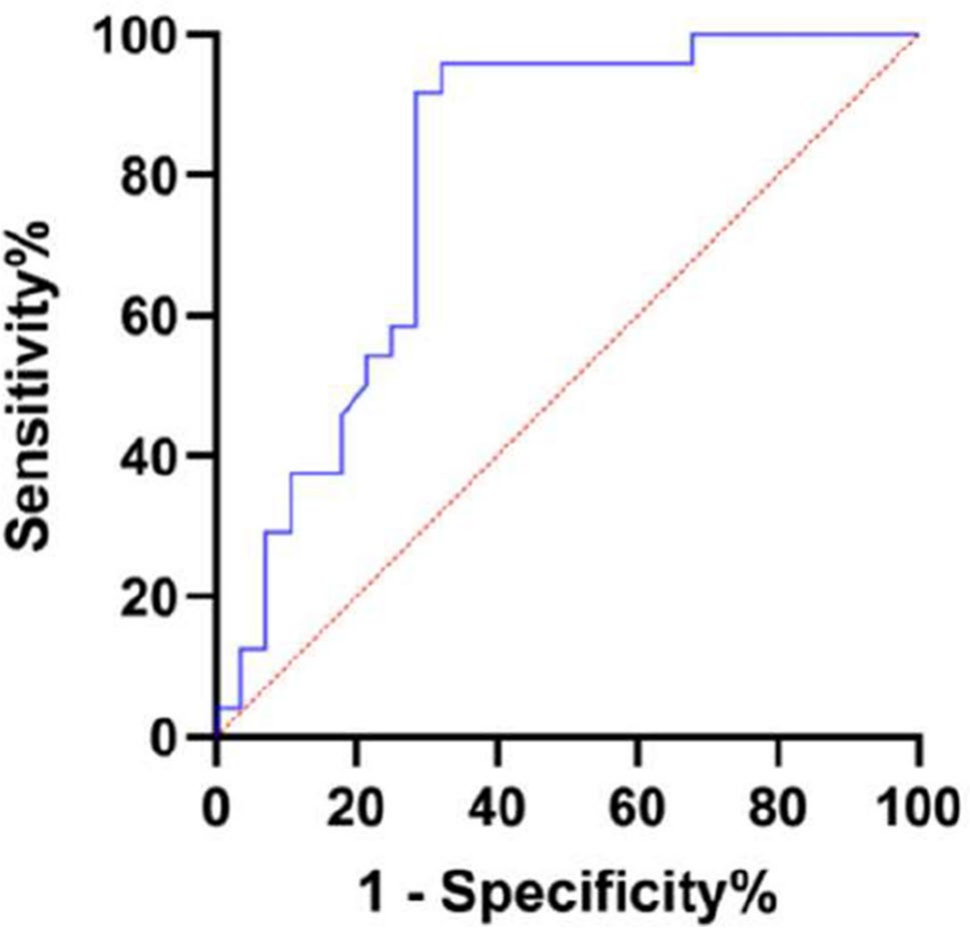
Diagnosis of PMR by CXCL10

**Fig. 4:**
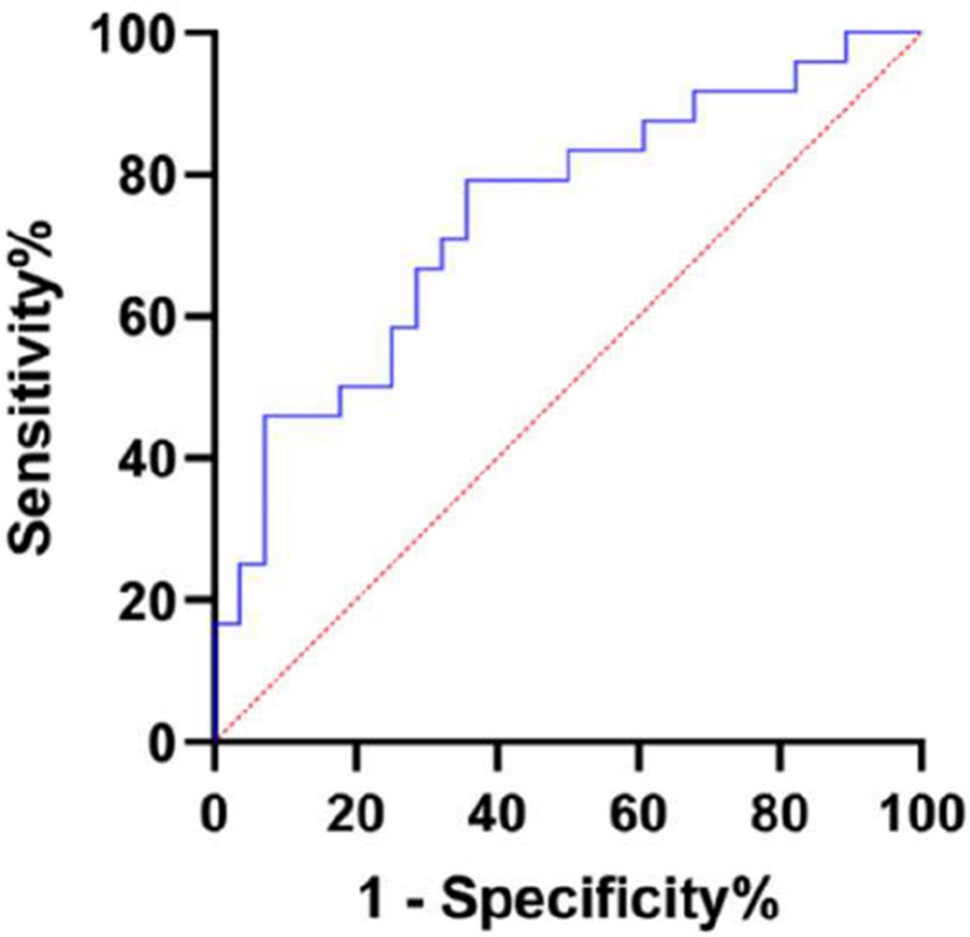
Differential diagnosis of PMR and RA by CXCL10

The ROC curve was constructed to evaluate the value of CXCL10 in the diagnosis and differential diagnosis of PMR. In the ROC curve for diagnosing PMR, the area under the curve is 0.797, sensitivity = 0.679, and specificity = 0.958. In the ROC curve for distinguishing PMR and RA, the area under the curve is 0.741, sensitivity = 0.643, and specificity = 0.792, as shown in Fig. 3 and Fig. 4.

## Discussion

PMR is a common inflammatory rheumatic disease prevalent in the elderly population, and it has been shown that pro-inflammatory cytokines, including interleukin 6 (IL-6), play an important role in its pathogenesis [2]. However, the mechanisms that maintain the persistence of inflammation are unclear. Chemokines have been found to play an important role in the pathogenesis of giant cell arteritis [11]. Therefore, the role of chemokines in the pathogenesis of isolated PMR was investigated in the present study. Serum chemokine protein chip screening of six PMR patients who were diagnosed and treated to obtain remission detected a significant difference in the expression of the chemokine CXCL10, which was validated by ELISA in 28 PMR patients after further expanding the samples, and found that the level of serum CXCL10 in active PMR patients was significantly higher than that of the remission PMR group, the RA group, and the normal control group. However, correlation analysis only found a correlation between CXCL10 and Fer, and no significant correlation was found with PMR clinical activity parameters such as ESR, CRP, and PMR-AS. ROC curves showed that the level of CXCL10 can be biomarker for diagnosis and differential diagnosis of PMR and RA.

CXCL10 is also known as IP-10 [(interferon (IFN)-γ-induced protein 10], and the corresponding receptor is CXCR3. CXCL10 is synthesized and secreted by IFN-γ-induced T-lymphocytes, monocytes, endothelial cells, fibroblasts, and others. The CXCL10-CXCR3 axis is involved in the process of inducing immune cell chemotaxis, and IFN-γ and TNF-α produced by Th1 cells can promote the secretion of CXCL10 by T cells, monocytes, and endothelial cells, etc., and CXCL10, in turn, recruits and activates CXCR3-expressing Th1 cells to form an amplified loop [3]. A study by Van der Geest et al. [6] also found significantly elevated serum CXCL10 levels in patients with active PMR, similar to the present study.

In this study, we found a significant positive correlation between CXCL10 levels and serum ferritin in PMR patients, which belongs to the acute time-phase response proteins and is elevated in acute inflammation, malignancy, autoimmune diseases, and infections [12]. Ferritin-like structures have also been found in various bacterial species. Bacteria use ferritin-like structures for detoxification of iron and for iron [13]. N T Baerlecken et al. had found that antibodies against the ferritin peptide, which can enhance the resistance in the defense against bacterial infections [14], were present in PMR patients [15]. In autoimmune diseases, pro-inflammatory cytokines such as TNF-α and IL-6 are caused it to be elevated [16].Therefore, it is hypothesized that PMR may be induced by bacterial infections, where cytokines stimulate the expression of ferritin genes [17] and the production of antiferritin antibodies in response to infection [18, 19]. CXCL10 may cause elevation of ferritin by inducing the release of pro-inflammatory factors from inflammatory cells. There was no significant correlation between serum CXCL10 levels and clinical activity parameters such as ESR, CRP, and PMR-AS in active PMR patients. A significant positive correlation between the pro-inflammatory cytokine IL-6 and clinical activity parameters of PMR, such as ESR and CRP, has been established [6]. And it was hypothesized that the main biological effect of CXCL10 as a chemokine is to transmit immune information and chemotaxis immune cells to migrate, but it is not a direct pro-inflammatory factor. It also requires expansion of the samples for in-depth study.

In this study, CXCL10 levels were found to be significantly higher in active PMR patients than in RA patients. It has been shown that CXCL10 levels are significantly elevated in synovial tissue and synovial fluid of RA patients [20], suggesting that CXCL10 is also involved in the development of RA. Chemokines occur as networks of biological effects, and one chemokine can act on multiple immune cells and participate in the pathogenesis of a variety of autoimmune diseases. The exact pathogenic role of CXCL10 in PMR versus RA and its differences need to be studied in depth, for example, by selecting seronegative and positive RA patients to study how differences in the chemokine CXCL10, to gain insight into the value of differential diagnosis with RA. It has been reported in the literature [21] that CXCL10 levels were significantly higher in RA patients than in normal controls, whereas no difference was seen in our data, which may be related to the long storage time of serum specimens in this study and the small sample size of RA used as a case–control group. However, significantly higher levels of CXCL10 in the PMR group than in the RA group were evident in this study. And the ROC curve for distinguishing PMR and RA, the area under the curve is 0.741, sensitivity = 0.643, and specificity = 0.792. It is suggested that the chemokine CXCL10 has the potential to be used as a differential diagnostic biomarker between PMR and RA.

In summary, PMR, as a chronic inflammatory disease, requires the joint participation of inflammatory factors and chemokines for the maintenance of chronic inflammation, and CXCL10 plays a role in the pathogenesis of PMR. The correlation between serum CXCL10 levels and serum ferritin is in line with the hypothesis that PMR may be induced by bacterial infections. And it is also worthwhile to conduct an in-depth study on whether CXCL10 can be used as a differential diagnostic marker from RA, especially seronegative RA.

## Supplementary Information

Below is the link to the electronic supplementary material.

Supplementary file1 (XLS 28 KB)

## Data Availability

All relevant data are within the paper. The data that support the findings of this study are available from the authors on reasonable request.
